# Book Reviews

**Published:** 1898-05

**Authors:** 


### Book Reviews.

DISEASES OF THE STOMACH. Their special pathology, diagnosis and treatment, with sections on anatomy, physiology, analysis of stomach contents, dietetics, surgery of the stomach, etc. In three parts. By JOHN C. HEMMETER, M. B., M. D., Philos. D., Clinical Professor of Medicine at the Baltimore Medical College; Consultant to the Maryland General Hospital, etc. Royal octavo, pp. xvii.—788. With illustrations, some in colors, and a lithograph frontispiece. Philadelphia: P. Blakiston, Son & Co., 1012 Walnut street. 1897.

The first part of this treatise is devoted to a consideration of the anatomy and physiology of the digestive organs, together with the methods and technics of diagnosis. The author briefly deals with the anatomy of the stomach in chapter one, and with its histology in chapter two. The latter chapter is chiefly an extract from Mr. Bensley's conclusions in his paper read to the Canadian Institute in 1896. Mr. Bensley's drawings with reference to the gland cells of the cat's stomach are both original and interesting. The small intestine forms the subject of the third chapter, and this is a section of the digestive tract that always possesses interest, because of the liability to cancerous changes at the junction of the pylorus and duodenum.

The physiology of digestion is taken up in the fourth chapter, together with methods and technics of diagnosis, and its consideration is continued during its several phases until the end of this part. In regard to technics, Hemmeter has some methods peculiar to himself, one of which he designates as Hemmeter's method for testing absorption, which he describes in the tenth chapter, page 93. The author is especially interesting in his description of the use of the stomach-tube, having himself devised some modifications in its accessories.

In the second part the author considers the therapy and materia medica of stomach diseases. He very properly lays great stress upon dietetic treatment of gastric diseases, giving elaborate tables of dietetics and amply prepared diet lists. The effects of cooking on food are not very well understood by the average cook, and it is desirable that persons suffering from stomachal diseases should derive the utmost benefit from a carefully arranged diet list and properly cooked food. Hemmeter gives due weight to these important branches of his subject. He also discusses at considerable length the uses and abuses of natural mineral waters in digestive disorders. The majority of laymen and only a moderate ratio of physicians give much consideration and less scientific thought to this important question. Fashion, fad, predilection—all these and many other equally unscientific reasons are allowed to govern the choice of springs, whereas this should be a subject of quite as much thought as any branch of therapy relating to gastro-intestinal disturbances.

The surgical treatment of organic gastric diseases is discussed in chapter seven, where pylorectomy is advocated in carcinoma and some other affections as the only operation likely to make definite cure or recovery of some duration possible. Haberkant's tables are given, and these merit careful study. The author next proceeds to consider the influence of gastric diseases upon other organs and on metabolism ; and in chapter nine he considers the blood and urine in stomach diseases.

The third part of this treatise, which comprises about one-half of the volume, is allotted to what the author calls the gastric clinic. In it he considers all the special diseases of the stomach, such as gastritis, gastric ulcer, tuberculosis and syphilis of the stomach, benign tumors, motor insufficiency, enteroptosis, neuroses of the stomach, etc., etc. Hemmeter will be found especially interesting in this part of his book, and he is to be credited with considerable originality in his treatment of the subjects, as well as skill in the form of presenting them. He has been careful to enumerate the bibliography of the various portions of his treatise, which is attached to the several chapters as the text progresses.

An index of authors and of subjects, both well prepared, brings to a close this exceedingly interesting and useful volume. It is the most elaborate treatise yet presented to the profession upon diseases of the stomach, and will easily become a text-book in colleges. Hemmeter, Einhorn and Ewald are the standard modern treatises upon the stomach and its diseases, but where only one can be afforded, the student or practitioner can make no mistake in choosing Hemmeter. The publishers have made it an attractive volume in its mechanical make-up.

---

The International Medical Annual and Practitioner's Index. A Work of Reference for Modern Practitioners. Sixteenth year. New York: E. B. Treat, 241–243 W. 24th street. 1898.

The sixteenth annual volume of the Medical Annual, which has just appeared, is on the same lines which have already met with the approval of hundreds of physicians the country over. Besides reviewing the progress of medical science from year to year, special

REPORT OF THE COMMISSIONER OF EDUCATION FOR THE YEAR 1895–96. Volume II. Containing Part II. Washington: Government Printing Office. 1897.

The second part of this report, which is comprised in Volume II., deals with education in Sweden and Iceland; typical institutions offering manual or industrial training; higher and secondary education in the United States; how agriculture is taught in Prussia and

France; industrial education in Germany, Austria and Switzerland; recent efforts in Europe for the advancement and improvement of agriculture; colleges endowed by congress for the benefit of agriculture and the mechanic arts; the Bertillon system as a means of suppressing the business of living by crime; art decorations in school rooms, together with various statistics and numerous concurrent matters of interest. It gives statistics of the schools of medicine in the United States, but as these are now two years old or more, they are of little use. From the foregoing synopsis the value of this volume to the educators of the country will become apparent.

---

A Clinical Text-Book of Surgical Diagnosis and Treatment. For Practitioners and Students of Surgery and Medicine. By J. W. Macdonald, M. D., Graduate in Medicine of the University of Edinburgh; Licentiate of the Royal College of Surgeons, Edinburgh; Professor of the Practice of Surgery and of Clinical Surgery in Hamline University, Minneapolis, etc. Royal octavo, pp. 798. With 328 illustrations. Philadelphia: W. B. Saunders, 925 Walnut street. 1898.

The contributions to surgical literature in tomes, systems and text-books of late have been so considerable, and from authors of greater or less renown, that the reason for a new treatise at this time must be very cogent to justify its issuance. This author, comparatively unknown to fame, has undertaken to answer two questions as the *raison d'etre* of his book. They are of such supreme importance as to embrace the whole field of medical and surgical literature. The first question, as stated by the author in his preface, is, "What is the disease or injury?" The second question is, "What is the proper treatment?"

The first chapter of the book is addressed to the general examination of patients, in which, without unfolding any novelty, a concise statement of essentials is given. The next chapter tells of examination of the vascular system, which well merits the reader's careful attention. Macdonald's handling of aneurism, particularly his differential diagnosis of special aneurisms, is admirable. It is one of the most important subjects for the young surgeon to study with care.

Injuries and diseases of the osseous system are next considered in the following order: fractures, dislocations, inflammations of bone, tumors of bone and deformities. Fractures and dislocations being the commoner and easier diagnosticated injuries, we pass without comment. Macdonald becomes interesting in his treatment of inflammations and tumors of bones. Of the latter he publishes some instructive illustrations, many of which are new. Of next importance are injuries and diseases of joints, that are also dealt with systematically, concisely and with a reasonable degree of scientific accuracy.

Under the general title of injuries and diseases of the digestive system, is considered almost every known disease or injury that can attack or befall the human economy from hare-lip to imperforate rectum. This section covers nearly 200 pages and is illustrated with many original engravings. The author is lucid in his treatment of most of these conditions. Take hernia, for instance, to which he devotes twenty pages, in which its anatomy is clearly set forth and the several operations in vogue—Championniere's, Macewen's, Bassini's, Halstead's—are clearly described and some good illustrations given to elucidate the text. Appendicitis is less elaborately dealt with, though Fowler's excellent colored plate, illustrating some of the changes observed in acute appendicitis, is introduced.

The genito-urinary system forms the subject of the next chapter, consisting of 110 pages. Naturally the general surgeon lays great stress upon stone in the bladder and the operations for its relief. Macdonald is no exception to the rule and this forms the chief point of interest in this section. Prostatitis, however, should be mentioned as receiving considerate and skilful attention.

We come now to injuries and diseases of the head, in which chapter is set forth modern methods of diagnosis and management. Cerebral tumors and epilepsy are but briefly considered, still it is always necessary to refer to monographs on these subjects, of which the recent work of Phelps is a type. Injuries, diseases and deformities of the spine next follow and these are well handled and admirably illustrated. However, here again one must consult treatises on orthopedic surgery for detailed information. A good chapter is written on diseases and injuries of the nerves, and another on diseases and injuries of the respiratory system, the latter being somewhat extensive in its dealings with the nose and throat. Next follow chapters on the diagnosis and treatment of syphilis, the diagnosis and treatment of tumors, diseases and injuries of the neck, and injuries and diseases of the breast, all of which, though short, contain much practical information.

The author next offers us a somewhat extended chapter on diseases and injuries of the female generative organs. In it he offers a few platitudes, presents some excellent drawings and illustrations, quotes the American Text-book of Gynecology on the technique of supravaginal amputation and hysterectomy and refers to Joseph Price and Bland Sutton, two excellent authorities, in regard to ectopic pregnancy. He very properly condemns the use of electricity as a means of destroying the life of the fetus before tubal rupture and asserts that nothing short of removal of the gestation sac is indicated. He is misleading and we think surgically unsound, however, when he declares that the patient may survive after rupture and the condition be palliated for days, weeks and months without surgical interference; yet in the next sentence he clears the atmosphere by asserting that the indication is to operate on all intraperitoneal cases at the earliest possible moment by abdominal section.

The final chapter deals with the Röntgen rays in surgical diagnosis and presents some very good illustrations. The book has very many excellent points, as we have indicated, and is a valuable contribution to the literature of surgery.

Biennial Report of the Department of Health of the City of Chicago for the Years 1895 and 1896. William R. Kerr, Commissioner of Health. Chicago: 1897.

This standard octavo volume comprises the reports of the health commissioner of Chicago for the years 1895 and 1896. It is divided into four parts with an aggregate of 440 pages. The first part embraces the reports of divisions and bureaus; the second part gives the vital statistics of the city of Chicago for the years mentioned; the third part is devoted to statistics and the fourth part to a chronological summary of Chicago mortality from 1851 to 1896, inclusive.

The reports of the division of contagious diseases is an interesting part of the work of the health board, and the volume contains some good illustrations. Like all municipal printing, however, it is not properly sub-divided, nor are the page headings of subjects given. Instead thereof, across the top of each page is printed "Report of the Department of Health of the City of Chicago." It should have been paged consecutively from beginning to end throughout the several parts; an index, then, would have completed an otherwise very creditable report.

---

A MANUAL OF OBSTETRICS. By A. F. A. KING, M. D., Professor of Obstetrics and Diseases of Women in the Medical Department of the Columbian University, Washington, D. C., and in the University of Vermont, etc. New (seventh) edition. Duodecimo, pp. 573, with 223 illustrations. Philadelphia and New York: Lea Brothers & Co., Publishers. 1898.

The appearance and exhaustion of edition after edition of this manual bespeaks in more emphatic terms than words of the hold it has taken upon both students and practitioners of the obstetric art. Dr. King builded better than he knew when he modestly put forth the first edition of his little book. It has grown steadily in favor, until there is scarcely a physician's office of any prominence throughout the breadth and length of the land in which one or more copies are not to be found. Its eminently practical character has commended it to the favor of practical physicians. We wish especially to invite attention to the excellent summary of the jurisprudence of midwifery presented in the last chapter of the book. This is a subject in which medical students usually obtain scant instruction. Obstetric teachers will do well to enlarge upon this topic.

---

PROCEEDINGS OF THE UNITED STATES VETERINARY MEDICAL ASSOCIATION. Session of 1897. Thirty-fourth annual meeting, held at Nashville, September 7, 8 and 9, 1897. Edited by the publication committee, W. L. Williams, Chairman, Cornell University, Ithaca, N. Y.

The importance of the science of veterinary medicine is well accentuated in the pages of this book. For thirty-four years the association has continued its annual meetings, advancing steadily from a comparatively small initiative until it has now become a great organization. Its members for the most part are men well educated in veterinary science, and this book of 272 pages is a testimonial of its scientific worth as a progressive association of veterinary surgeons.

Transactions of the National Confederation of State Medical Examining and Licensing Boards. Seventh annual meeting held in Philadel phia, Pa., May 31, 1897. A. Walter Suiter, M. D., Secretary.

This brochure of seventy-eight pages contains the proceedings of the associated state medical examining boards during the seventh annual meeting, held at Philadelphia, Pa., May 31, 1897. It is a carefully prepared report of the actual business of the confederation, including addresses and papers read at the meeting, together with discussions had thereon. To these are added the constitution, the addresses of secretaries of state examining boards, and some notes of interest and information by the secretary of the confederation. While the book is full of interest, the preliminary report of the committee on minimum standards will attract chief attention. At the forthcoming meeting (Denver, June 6, 1898,) this committee will present an elaborate and well-digested report, which will claim the consideration of all thoughtful men interested in maintaining advanced standards in medical education.

The publication of this brochure in separate form is to be commended and every college teacher and state examiner throughout the country should obtain a copy.

---

Transactions of the Oregon State Medical Society. Twenty-fourth annual meeting, held at Portland, Oregon, June 8 and 9, 1897. William F. Amos, M. D., Secretary.

When we reflect that this is the report of the twenty-fourth annual meeting of the Oregon state medical society, the thought dawns upon us that the young and ambitious West is no longer youthful, but is getting on well toward maturer years. This modest little pamphlet of eighty-two pages contains an interesting record of two well-spent days. We congratulate our Oregon friends in having reached so substantial a stage in their medical progress.

---

Twenty-third Annual Report of the Secretary of the State Board of Health of the State of Michigan for the fiscal year ending June 30, 1895. Lansing: Robert Smith Printing Co. 1896.

This volume contains the report of the operations of this efficient health board between July 1, 1894 and June 30, 1895. It is replete with excellent material that will prove of interest to sanitarians, public health officers and all interested in preventive medicine. These reports are somewhat slow in making their appearance. It would seem as though their value might be increased by issuing them within the year subsequent to the period they cover. The secretary, Dr. Henry B. Baker, is one of the most efficient executive officers of any board of health within the union, and his work always tells influentially upon the prevention of disease.

---

Transactions of the American Microscopical Society. Twentieth annual meeting, held at Toledo, O., August 5, 6 and 7, 1897. William C. Krauss, M. D., Secretary.

This excellent report of the transactions of this scientific society is a credit to the members and an addition of decided merit to the literature of microscopy. It contains, too, some handsome plates. The volume is somewhat less in size than last year, but it is none the less indicative of excellent work. The only regret is that it is not issued in boards, paper covers being very unsatisfactory for scientific works of reference.

